# Predictors of Biased Self-perception in Individuals with High Social Anxiety: The Effect of Self-consciousness in the Private and Public Self Domains

**DOI:** 10.3389/fpsyg.2017.01126

**Published:** 2017-07-04

**Authors:** Henrik Nordahl, Alice Plummer, Adrian Wells

**Affiliations:** ^1^Department of Psychology, Norwegian University of Science and TechnologyTrondheim, Norway; ^2^Division of Psychiatry, St. Olav’s University HospitalTrondheim, Norway; ^3^Renal Services, St. George’s University Hospitals NHS Foundation TrustLondon, United Kingdom; ^4^Division of Psychology and Mental Health, School of Health Sciences, Faculty of Biology, Medicine and Health, University of Manchester, Manchester Academic Health Science CentreManchester, United Kingdom; ^5^Greater Manchester Mental Health NHS Foundation TrustPrestwich, United Kingdom

**Keywords:** biased self-perception, social anxiety, social phobia, self-focused attention, self-consciousness

## Abstract

“Biased self-perception,” the tendency to perceive one’s social performance as more negative than observers do, is characteristic of socially anxious individuals. Self-attention processes are hypothesised to underlie biased self-perception, however, different models emphasise different aspects of self-attention, with attention to the public aspects of the self being prominent. The current study aimed to investigate the relative contribution of two types of dispositional self-attention; public- and private self-consciousness to biased self-perception in a high (*n* = 48) versus a low (*n* = 48) social anxiety group undergoing an interaction task. The main finding was that private self-consciousness explained substantial and unique variance in biased negative self-perception in individuals with high social anxiety, while public self-consciousness did not. This relationship was independent of increments in state anxiety. Private self-consciousness appeared to have a specific association with bias related to overestimation of negative social performance rather than underestimation of positive social performance. The implication of this finding is that current treatment models of Social anxiety disorder might include broader aspects of self-focused attention, especially in the context of formulating self-evaluation biases.

## Introduction

A core feature of Social anxiety disorder (SAD) or social phobia is a pronounced fear of negative evaluation. To explore factors contributing to inflated social fears, researchers have investigated observer and self-ratings of social performance. “Biased self-perception” refers to the tendency of high socially anxious individuals to perceive their social performance as more negative than observers do, a bias that has been consistently demonstrated and that is more salient in socially anxious individuals than controls (e.g., Rapee and Lim, 1992; Stopa and Clark, 1993; Alden and Wallace, 1995; Norton and Hope, 2001; Moscovitch and Hofmann, 2007; Brozovich and Heimberg, 2011; Cody and Teachman, 2011; Zou and Abbott, 2012; Gavric et al., 2017). Biased self-perception is thought to be an important maintenance factor in SAD and should therefore be conceptualized and addressed in treatment. However, the factors that might underlie and contribute to biased self-perception have not been extensively explored.

Cognitive models of social anxiety (e.g., Clark and Wells, 1995; Rapee and Heimberg, 1997; Hofmann, 2007; Heimberg et al., 2010) give a central role to biased self-perception and attention styles in disorder maintenance. According to the Clark and Wells (1995) model, negative assumptions that relate to performance failure and catastrophic consequences of exhibiting anxiety symptoms are activated when a socially anxious individual enters a social situation, and this leads to a shift to internal focused attention where the individual focuses on an inner image of the self from an observer vantage point (“the observer perspective”). In this image, signs of anxiety and failed performance are subjectively seen as exaggerated and conspicuous, and the individual erroneously believes this image accurately represents how she or he appears to others (Wells, 1997). In support of the Clark and Wells (1995) model, taking the observer perspective has been found to increase anxiety during social situations and to be associated with more negative self-ratings of performance (Vassilopoulos, 2005; Stopa and Jenkins, 2007; Makkar and Grisham, 2011; Nordahl et al., 2016), and greater discrepancy between self- and observer ratings of performance (Hirsch et al., 2003, 2004). Thus, in line with the Clark and Wells (1995) model, biased self-perception could result from taking an inner focus on representations of the publicly observable aspects of self in social situations. However, this may not be the only or predominant self-attention process involved.

In a wider context, elevated self-focused attention has been demonstrated in most forms of psychopathology (Ingram, 1990) and has been assessed at a trait-level as private and public self-consciousness (Fenigstein et al., 1975). Private self-consciousness is the tendency to turn attention onto thoughts, feelings and attitudes (e.g., “I’m always trying to figure myself out”) whilst public self-consciousness assesses the tendency to focus on publicly observable aspects of self (e.g., “I’m concerned about the way I present myself”). Private self-consciousness in particular has been formulated in the metacognitive model (Wells and Matthews, 1994, 1996) as a marker of a transdiagnostic syndrome of repetitive negative thinking (i.e., worry and rumination) and attentional monitoring that causes psychological disorders. Specifically, excessive self-directed attention on thoughts is considered a central type of attention leading to unhelpful biases in self-regulation. Thus, allocating attention to private (repetitive ideational) aspects of self may contribute to negative self-evaluations more than attending to publicly observable aspects of self.

We therefore aimed to investigate the relative and unique contributions of public- and private self-consciousness to biased self-perception in a high and also a low social anxiety group undergoing an interaction task. High socially anxious individuals have been found to be a valid analogue for SAD (Stopa and Clark, 2001), and it has been argued that analogue research provides a suitable framework for testing information-processing biases that may maintain SAD, thus facilitating progress in the field (Hirsch and Clark, 2004). We included a low social anxiety group in the analysis because we cannot assume that the relationships between self-attention and biased self-perception are linear or have the same pattern of associations across the spectrum of social anxiety. We also measured state anxiety because it is important to control for anxiety symptom levels that may explain the relationship between self-attention and bias in self-perception.

Our hypotheses were tested in the context of a social interaction task and primarily concerned biases in perception of negative responses (i.e., those with social anxiety over-estimate these) but we also explored if there were attention correlates of the underestimation of positive behaviours. Our hypotheses were as follows; (i) the high socially anxious group will present with a significantly greater bias than the low-socially anxious group showing an overestimation of their negative behaviours and an underestimation of their positive behaviours; (ii) Consistent with the Clark and Wells (1995) model, public self-consciousness will explain variance in biased self-perception; (iii) Consistent with the Wells and Matthews (1994) model, private self-consciousness will explain additional variance in biased self-perception when controlling for public self-consciousness; (iv) Public and private self-consciousness will not predict biased self-perception in the low socially anxious group as these individuals don’t shift their attention towards self-processing in social situations.

## Materials and Methods

### Participants

Participants were recruited from the University of Manchester by a mass email inviting them to participate in the experiment. A total of 665 students responded to the mass email, who were then emailed a copy of the FNE (Watson and Friend, 1969) and were asked to report their age and gender. Participants had to fulfil the following inclusion criteria: (a) minimum age of 18 years, and (b) score of 7 and lower for the low social anxiety group, and of 22 and higher for the high social anxiety group on the FNE (Stopa and Clark, 2001). Of the 370 students who returned the FNE, 151 met the inclusion criteria, and 96 volunteered to participate in the experiment. Informed consent was obtained from everybody that volunteered. Hence, the sample consisted of 96 students, of which 56 (58.3%) were female and 40 (41.7%) male. On the basis of their FNE scores, a high and low social anxiety group were formed with the constraint that there were equal numbers of men and women in each group. Thus, each of the groups contained 48 participants: 28 females and 20 males. The mean age for the low FNE group was 22.48 (*SD* = 5.07) and 21.67 (*SD* = 4.93) for the high FNE group (*p* = 0.428). The mean FNE scores were 3.79 (*SD* = 2.27) for the low FNE group and 25.79 (*SD* = 2.56) for the high FNE group.

### Measures

The Behavioural Checklist (BCL; Papageorgiou and Wells, 2002) is a modified version of Lewinsohn et al.’s (1980) scale, and assesses participants’ social performance during interaction tasks. The scale has two subscales and consists of 10 negative (e.g., “boring”) and 15 positive (e.g., “confident”) items, which are rated on a 7-point rating scale from 1 (“not noticeable”) to 7 (“very noticeable”). Negative scores range from 10 to 70, with higher scores indicating worse performance. Positive scores range from 15 to 105, whereby a higher score indicates better performance. Papageorgiou and Wells (2002) used the BCL in order to obtain both self-ratings of participants’ social performance (BCL-1) and also other-ratings of participants’ performance (BCL-2). They observed good internal consistency, with a Chronbach’s alpha of 0.88 (negative subscale) and 0.94 (positive subscale) for the BCL-1, and 0.78 (negative subscale) and 0.93 (positive subscale) for the BCL-2. In the current study, the Cronbach’s alpha for the BCL-1 was 0.83 (negative subscale) and 0.78 (positive subscale). For the BCL-2, the Cronbach’s alpha was 0.86 (negative subscale) and 0.91 (positive subscale).

The Self-Consciousness Scale (SCS; Fenigstein et al., 1975) consists of 23 items measuring social anxiety and two dimensions of self-consciousness. For the current study, we used the two subscales measuring self-consciousness: Public Self-Consciousness assesses the tendency to view oneself from the perspective of the social world and reflects a concern for one’s appearance, social behaviour, and the impression one makes upon others. This subscale consists of seven items. Private Self-Consciousness, which assess the tendency to reflect upon oneself, and to attend to one’s moods, motives, and cognitive processes. This subscale consists of 10 items. Respondents are asked to rate how characteristic each item is of them on a 5-point scale ranging from 0 (“extremely uncharacteristic”) to 4 (“extremely characteristic”). The Public Self-Consciousness subscale scores range from 0 to 28, while the Private Self-Consciousness subscale ranges from 0 to 40. The internal consistency has been reported as acceptable and is 0.79 for the public self-consciousness subscale, and 0.69 for the private self-consciousness subscale (Scheier and Carver, 1985). In the current study, the Cronbach’s alpha was 0.88 for the public self-consciousness subscale, and 0.74 for the private self-consciousness subscale.

The Spielberger State-Trait Anxiety Inventory, State anxiety subscale (STAI-S; Spielberger et al., 1983) is a 20 item self-report questionnaire employed as an index of anxiety experienced by an individual at a given moment in time. Respondents are asked to rate how much they agree with each of the statements on a four-point scale. Total scores range from 20 to 80 points, with higher scores indicate more intense feelings of state anxiety, “right now, that is, at this moment.” The STAI-S was selected for this study as it is sensitive to changes in transitory anxiety. The STAI-S has good psychometric properties, with a median Cronbach’s alpha of 0.92 (Spielberger et al., 1983). In the current study, the internal consistency was good both before (α = 0.94) and after (α = 0.96) the interaction task.

### Procedure

This study was carried out in accordance with the recommendations of the University of Manchester Committee on the Ethics of Research on Human Beings with written informed consent from all subjects. All subjects gave written informed consent in accordance with the Declaration of Helsinki. The protocol was approved by the University of Manchester Committee on the Ethics of Research on Human Beings. A postgraduate student of clinical psychology was appointed on a voluntary basis as a confederate for the experiment. The confederate was blind to the conditions (high versus low FNE), so as not to confound the results.

Upon arrival, all participants were given an information sheet and signed the consent form. Participants were then asked to complete the STAI-S (Spielberger et al., 1983) and the SCS (Fenigstein et al., 1975). Next, participants were provided with the following experimental instructions: *I would like you to hold a brief, 5-min conversation with a colleague, who will shortly be entering the room. You may talk about whatever you like, but please do not talk about the experiment. Please make conversation about the kind of things that you normally would in social situations. So it is important that you start the conversation and try to keep it going, until I tell you to stop.*” Participants were then given 1 min to contemplate the forthcoming interaction task, after which the confederate entered the room. The confederate was instructed to engage in a friendly manner and to let the participant do most of the talking. A set of questions (e.g., “What are you studying?” and “What things do you enjoy doing?”) was provided in the event of pauses in the conversation of 20 s or more, or if the participant started to talk about the experiment itself. At the end of the 5 min, the confederate left the room, and the participant was asked to complete the STAI-S (Spielberger et al., 1983) and the BCL-1 (Papageorgiou and Wells, 2002) for self-rated performance. At the same time, the confederate used the BCL-2 (Papageorgiou and Wells, 2002) to rate the participants’ social performance. All participants were appropriately debriefed on completion of the experiment.

### Overview of Data Analyses

To calculate biased self-perception, we subtracted the BCL-1 score (self-rating) from the BCL-2 score (other-rating) for both the negative and the positive subscale. A greater score denotes a greater discrepancy, with a negative score for negative behaviours indicating an overestimation of negative behaviours by the participant and a negative score on positive behaviours indicating an overestimation of positive behaviours. We calculated a change score in state anxiety by subtracting STAI-S pre from STAI-S post interaction task.

Independent samples *t*-tests were used to compare the high and low FNE group on negative and positive biased self-perception, change in state anxiety and public and private self-consciousness. Then we ran bivariate correlational analyses to investigate the relationship between negative and positive biased self-perception, change in state anxiety, and public and private self-consciousness within the low and the high FNE group.

Hierarchical linear regression analyses were planned; one for positive and one for negative biased self-perception in each group to explore which variables predicted biased self-perception. We controlled for gender and age in the first step, change in state anxiety in the second step, public self-consciousness was entered on the third step, and private self-consciousness was entered in the final step.

## Results

### Group Comparisons

The high FNE group scored significantly greater than the low FNE group on negative and positive biased self-perception, indicating that they overestimated their negative social performance and underestimated their positive social performance compared to the low FNE group. The high FNE group also showed a significantly greater change in state anxiety, indicating greater subjective anxiety levels as a result of the interaction task. The public and private self-consciousness scores demonstrated that the high FNE group scored significantly higher indicating a greater tendency to be self-conscious in social situations. The group comparisons are presented in **Table 1**.

**Table 1:** Group comparison between the high and low FNE groups; means, standard deviations and *t*-values.

	Low FNE (*n* = 48)	High FNE (*n* = 48)	*t*-value
Biased self-processing, negative	–6.98 (8.82)	–18.71 (14.11)	4.884^∗∗^
Biased self-processing, positive	6.54 (19.11)	19.98 (14.59)	–3.584^∗∗^
State anxiety change	0.10 (4.63)	3.29 (9.40)	–2.107^∗^
Public self-consciousness	12.29 (4.95)	21.98 (3.06)	–11.529^∗∗^
Private self-consciousness	20.48 (5.36)	26.17 (4.97)	–5.394^∗∗^


*^∗^*p* < 0.05, ^∗∗^*p* < 0.01.*

### Correlational Analyses

In the low FNE group, there was a significant negative association between negative and positive biased self-perception, indicating that an overestimation of negative social performance was associated with an underestimation of positive social performance among the low-socially anxious individuals. None of the other variables were associated with each other.

In the high FNE group, the same significant associations between negative and positive biased self-perception were observed. In addition, negative biased self-perception was significantly negatively associated with private self-consciousness. The other variables were not associated with each other. The correlations are presented in **Table 2**.

**Table 2:** Bivariate correlations for negative and positive biased self-perception, change in state anxiety, public self-consciousness and private self-consciousness for both the low (*n* = 48) and the high (*n* = 48) FNE group.

			2	3	4	5
**Low**	1	Biased self-perception, negative	–0.602^∗^	–0.165	–0.158	–0.130
	2	Biased self-perception, positive		0.063	–0.039	0.077
	3	State anxiety change			0.033	–0.088
	4	Public self-consciousness				0.284
	5	Private self-consciousness				
**High**	1	Biased self-perception, negative	–0.443^∗^	0.157	–0.045	–0.382^∗^
	2	Biased self-perception, positive		–0.140	–0.120	0.044
	3	State anxiety change			0.028	0.008
	4	Public self-consciousness				0.378^∗^
	5	Private self-consciousness				


*^∗^*p* < 0.01.*

### Regression Analyses

Our primary goal was assessing predictors of negative behaviour biases in the high social anxiety group. After controlling for age, gender, anxiety and public self-consciousness, private self-consciousness made a significant independent contribution to overestimation of negative behaviours, explaining an additional 17% of the variance. The negative β-value indicates a positive relationship between private self-consciousness and greater negative biased self-perception. The results of this analysis for negative biased self-perception in the high FNE group are presented in **Table 3**. We had planned to run exploratory regressions predicting bias in positive behaviours but as there were no bivariate correlates of such bias we did not proceed with these analyses.

**Table 3:** Statistics for each step of the regression and betas on the final step with negative biased self-perception as dependent variables and gender/age, change in state anxiety, public self-consciousness and private self-consciousness as predictors in the high FNE group (*n* = 48).

Step		*F* cha	*R*^2^ cha	β	*t*
1		0.512	0.022		
	Gender			–0.15	–0.966
	Age			–0.01	–0.062
2		0.842	0.018		
	Gender			–0.13	–0.808
	Age			–0.00	–0.027
	State anxiety Δ			0.14	0.917
3		0.088	0.002		
	Gender			–0.13	–0.802
	Age			–0.01	–0.079
	State anxiety Δ			0.14	0.916
	SCS public			–0.05	–0.296
4		8.854	0.167		
	Gender			–0.18	–01.176
	Age			–0.02	–0.147
	State anxiety Δ			0.13	0.936
	SCS public			0.12	0.818
	SCS private			–0.44	–2.976^∗∗^


*^∗∗^*p* < 0.01.*

## Discussion

As predicted, we found that individuals high in social anxiety overestimated their negative behaviours and underestimated their positive behaviours in a social interaction task when compared with individuals with low social anxiety. They also reported greater increments in state anxiety. The main finding of this study was that private self-consciousness explained substantial and unique variance in biased self-perception in individuals with social anxiety, while public self-consciousness did not. Private self-consciousness did not appear to have a general effect on biased self-perception, but seemed to be specifically related to overestimation of negative social performance rather than underestimation of positive social performance. At the bivariate level, these relationships appear to be specific to groups of individuals with high levels of social anxiety as they did not appear in the low anxiety group.

This finding suggests that it may be useful to examine the multifaceted nature of self-focused attention as it relates to underlying biases in self-processing in disorders such as social anxiety. Whilst cognitive models (e.g., Clark and Wells, 1995) have emphasised focusing on public aspects of self-processing, and change in this domain seems to be important for treatment outcome (Gregory and Peters, 2017), this may not be the primary dimension of attention that contributes to overestimates of negative aspects of performance. The results of the current study suggest that such biases may be linked more uniquely to self-attention tendencies that prioritise thoughts rather than appearance. This finding is consistent with a metacognitive conceptualisation of psychological disorders (Wells and Matthews, 1994) in which psychological dysfunction is linked to excessive attention to thought activity, and is supported by a study by Nordahl et al. (2016) who found that positive metacognitive beliefs, (i.e., beliefs about the advantages of worrying), predicted negative self-evaluation of performance above the observer perspective in SAD patients. Similarly, positive metacognitive beliefs are associated with post-event processing (rumination) in high socially anxious individuals which is thought to maintain biased self-evaluations (Gavric et al., 2017). Thus, while self-attention in the public domain may be important for some cognitive biases in social anxiety (e.g., negative self-evaluation; Nordahl et al., 2016), self-attention in the private domain and its underlying correlates may be an important but overlooked contributor to other cognitive biases in social anxiety.

The implication of this study is that cognitive models of social anxiety might benefit by including a wider range of attentional factors. Not only prioritising the publicly observable domains of self but also considering the role of attention focused on cognitive events and the effect they might have in increasing biased negative perceptions of self. Interestingly, Cognitive behavioural therapy seems to effectively reduce self-focused attention (Gregory and Peters, 2017), but this improvement seems to be gained in the public- rather than in the private domain (Lundh and Öst, 2001; Bögels, 2006). It is therefore possible that conceptualization and treatment of social anxiety and its associated biases could be improved by also directly addressing self-attention in the private domain. In the metacognitive approach (Wells and Matthews, 1994), different aspects of self-processing are directed by underlying metacognitive beliefs (i.e., beliefs about cognition), and there is some evidence suggesting that metacognitive belief change may be a stronger predictor of symptom improvement than cognitive belief change (Nordahl and Wells, 2017; Nordahl et al., 2017). In a recent study, Vogel et al. (2016) treated social phobia patients with four weekly sessions of the Attention Training Technique (ATT; Wells, 1990) and four weekly sessions of Situational Attentional Refocusing (SAR; Wells, 2000). These techniques build on the metacognitive model and aim to address the excessive cognitive regulatory processes of excessive self-focused attention rather than the content of cognition. Forty-six per cent of the total sample no longer met DSM-IV criteria for social phobia at post intervention even though there was no focus on challenging of thoughts or the accuracy of the self-image. This data suggests that metacognitive techniques that directly attenuate self-attention and increase attention control could provide a new line of treatment for social anxiety. Further research should evaluate the relative effects of directly targeting private- and public self-consciousness on self-perceptions in socially anxious individuals.

There are several weaknesses to our study that must be considered in the context of interpreting the data. First, we used only one observer to rate the social performance of the participants, and no inter-rater correlations could be determined. Second, we did not measure safety behaviours which have been suggested to contaminate social situations and increase self-focused attention (Clark and Wells, 1995) as supported by recent reviews (e.g., Piccirillo et al., 2015). The use of safety behaviours could therefore be an important factor to control for when investigating the relative contribution of public- and private self-consciousness in the discrepancy between observer and self-ratings of social performance in future studies.

## Conclusion

Private self-consciousness was associated with biased negative self-perception in socially anxious individuals, while public self-consciousness was not. It predicted an overestimation of negative self-judgements when anxiety level was controlled. The implication of this finding is that current treatment models of SAD might include broader aspects of self-processing when formulating the influence of attention on cognitive biases and may move towards a broader set of strategies for modifying different aspects of self-attention. For example, during CBT the therapist provides the patient with video feedback to correct the content of the observer image (public self-consciousness), so that the impression of the self may be more accurate. However, the current data support a different emphasis in which the therapist should help the individual to discover that focusing on inner images (irrespective of content) should be reduced, an approach which is more commensurate with metacognitive therapy (Wells, 2009).

## Author Contributions

All authors listed have made a substantial, direct and intellectual contribution to the work, and approved it for publication.

## Conflict of Interest Statement

The authors declare that the research was conducted in the absence of any commercial or financial relationships that could be construed as a potential conflict of interest.
